# Navigating Post-Traumatic Osteoporosis: A Comprehensive Review of Epidemiology, Pathophysiology, Diagnosis, Treatment, and Future Directions

**DOI:** 10.3390/life14050561

**Published:** 2024-04-26

**Authors:** Matthew B. Weiss, Shoaib A. Syed, Harris Z. Whiteson, Rahim Hirani, Mill Etienne, Raj K. Tiwari

**Affiliations:** 1 School of Medicine, New York Medical College, Valhalla, NY 10595, USAhwhiteso@student.nymc.edu (H.Z.W.); rhirani2@student.nymc.edu (R.H.); mill_etienne@nymc.edu (M.E.); 2 Graduate School of Biomedical Sciences, New York Medical College, Valhalla, NY 10595, USA; 3 Department of Neurology, New York Medical College, Valhalla, NY 10595, USA

**Keywords:** post-traumatic osteoporosis, biomineralization, hormonal imbalances, disuse osteoporosis, clinical management

## Abstract

Post-traumatic osteoporosis (PTO) presents a significant challenge in clinical practice, characterized by demineralization and decreased skeletal integrity following severe traumatic injuries. This literature review manuscript addresses the knowledge gaps surrounding PTO, encompassing its epidemiology, pathophysiology, risk factors, diagnosis, treatment, prognosis, and future directions. This review emphasizes the complexity of the etiology of PTO, highlighting the dysregulation of biomineralization processes, inflammatory cytokine involvement, hormonal imbalances, glucocorticoid effects, vitamin D deficiency, and disuse osteoporosis. Moreover, it underscores the importance of multidisciplinary approaches for risk mitigation and advocates for improved diagnostic strategies to differentiate PTO from other musculoskeletal pathologies. This manuscript discusses various treatment modalities, including pharmacotherapy, dietary management, and physical rehabilitation, while also acknowledging the limited evidence on their long-term effectiveness and outcomes in PTO patients. Future directions in research are outlined, emphasizing the need for a deeper understanding of the molecular mechanisms underlying PTO and the evaluation of treatment strategies’ efficacy. Overall, this review provides a comprehensive overview of PTO and highlights avenues for future investigation to enhance clinical management and patient outcomes.

## 1. Introduction

Bony injury at single sites due to modest trauma promotes reparative mechanisms including biomineralization to help repair the damaged structures. However, patients who have undergone complex traumatic injuries may develop demineralization, loss of bone mass, and decreased skeletal integrity, so-called post-traumatic osteoporosis (PTO) [1,2,3]. Advances in intensive care medicine have contributed to the increased survival of patients with previously terminal traumatic injuries, including those with polytraumas from high energy collision, burns, explosions, crush injuries, falls from height, and catastrophic events—increasing the incidence of this progressively debilitating pathology [4,5,6].

However, a comprehensive review addressing the spectrum of etiologies for post-traumatic osteoporosis (PTO), encompassing both diagnosis and management, is currently lacking in the literature. The existing literature primarily focuses on site-specific osteoporosis secondary to traumatic injury. Thus, we aim to provide a comprehensive review of the causes of osteoporosis secondary to trauma, while also delineating clinical diagnostic approaches, management strategies, and prospects for future research endeavors.

## 2. Epidemiology

Osteoporosis, the chronic and progressive loss of bone mass, affects approximately 12% of men and 30% of women worldwide, and leads to an increased risk of fracture, among other pathologies [7]. It is estimated that by 2025, approximately 3 million fractures per year will be ascribed to osteoporosis in the United States [8]. In the European Union, it is projected that the annual osteoporotic fracture rate will increase by 25% between 2019 and 2034 [9]. The estimated prevalence in Japan in 2021 was 15 million individuals, and with their aging population this is set to increase [10,11,12,13]. This risk of fracture increases with age and severity of disease, and notably about half of all women greater than age 50 will experience an osteoporosis-related fracture in their lifetime [14]. Due to the paucity of literature, epidemiological reviews specifically on PTO have been limited.

Fractures can be severely debilitating to patients, especially those that interfere with activities of daily living and quality of life. These include injuries of the hip, including femoral head or pelvic fractures, and injuries of the knee, including distal femoral and proximal tibial fractures. Up to 20% of patients with hip fractures will require long term care, and mortality estimates for patients with hip fractures are 10–45% [8,14,15,16]. Patients with spinal cord injury (SCI) must also be considered, as an underuse of extremities secondary to disability and dysfunction due to diminished innervation can cause muscle atrophy and osteoporosis. In these patients, incidence rates of lower extremity fracture have been reported to range from 1 to 34%, and for complete SCI up to a 50% fracture rate has been reported [17,18,19,20,21].

Osteoporosis is also a commonly under-diagnosed and under-treated disease, as 75% of women and 90% of men who are considered to be at high risk for osteoporosis do not undergo further workup, and more than 75% of patients with osteoporosis are not treated [22]. In addition, there is a high monetary burden on the US healthcare system, with estimates of up to USD 25.3 billion in cost for 2025 [7]. In the European Union, the burden of fragility fractures was approximated to be EUR 57 billion [9].

## 3. Pathophysiology

**Figure 1:**
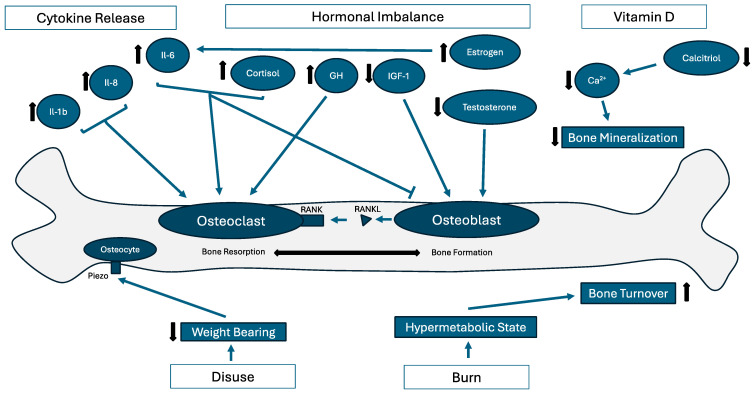
Mechanistic representation of processes involved in post-traumatic osteoporosis.

Post-traumatic osteoporosis can be caused by a wide range of traumatic processes, including burns and musculoskeletal injury, and is thus complex in nature (Figure 1) [2,3]. However, on the cellular and molecular levels, dysregulation of the biomineralization process takes a similar form across many diseases [23,24]. Biomineralization is a dynamic process of bone resorption by osteoclasts and formation by osteoblasts, intricately regulated by hormones and cytokines in the bone microenvironment. In polytrauma cases, an increase in cytokines interleukin (IL)-1β and IL-8 has been shown, as well as an increased IL-6/IL-10 ratio [25,26]. IL-1β is known to play a role in mediating osteoclastogenesis, osteoclast differentiation, and receptor activator in NF-kβ ligand (RANKL) expression [27,28,29,30,31]. IL-8 is a regulator of osteoclastogenesis as well and an inducer of nuclear factor of activated T cells 1 (NFATc1) activation by receptor activator of NF-kβ (RANK) [29,31]. IL-6 is known to upregulate osteoclastogenesis and downregulate osteoblastogenesis [32]. Increases in osteoclastogenesis and subsequent differentiation as well as a decrease in osteoblastogenesis lead to a catabolic state in the bone wherein resorption outcompetes synthesis, leading to demineralization and osteoporosis.

Glucocorticoid-induced osteoporosis is the most common form of iatrogenic osteoporosis [33,34], and glucocorticoids may play a role in PTO as well. The mechanisms by which glucocorticoids induce osteoporosis are multifaceted, involving direct effects of osteoblast and osteoclast activity, as well as regulating local concentrations of relevant growth factors (e.g., IGF-1) and cytokines (e.g., RANKL) [33]. A macaque model has shown an approximately 6-fold increase in cortisol levels after polytrauma and hemorrhage compared to hemorrhage alone [35], and an increase in glucocorticoid receptor β in the lungs of a polytrauma murine model has been shown [36], although more evidence is required to elucidate the role of glucocorticoids in PTO.

Other hormonal regulators of bone growth include growth hormone (GH) and insulin-like growth factor 1 (IGF-1), which are increased and decreased, respectively, in critically ill patients after trauma [37,38]. In vitro studies have shown that GH stimulates osteoclast differentiation and activation, causing bone resorption [39,40], and IGF-1 induces osteoblast differentiation and maintains bone mass [41,42]. Estrogen has been shown to be increased 10- to 15-fold from baseline after burn injury in females, but the data are conflicting for the male population [43,44,45]. These extremely elevated levels may upregulate the production of IL-6 [46]. Testosterone, however, has been shown to be increased in a range of traumas including burn, sepsis, and shock [45,47,48,49,50], and leads to increased osteoblast differentiation [51,52,53,54,55]. After trauma, these hormonal changes may perturb bone density homeostasis. Progesterone and parathyroid hormone are also regulators of bone homeostasis [56,57], although there is a lack of evidence of their roles in PTO.

A recent study found low (<30 ng/mL) vitamin D levels in 76% of patients in an inpatient rehabilitation facility, with diagnoses including neurologic injury and polytrauma [58]. Vitamin D and its active metabolite (1,25-dihydroxycholecalciferol) calcitriol are well documented to increase calcium and phosphate absorption in the gastrointestinal tract, increase calcium reabsorption by the kidney, and both have osteoblastogenic and osteoclastogenic effects on bone [59]. Thus, low levels of vitamin D may lead to low serum calcium levels, ultimately impeding biomineralization, although more research is needed before a conclusion may be drawn.

A related mechanism must be discussed, wherein disuse osteoporosis may be induced secondary to prolonged bed rest or trauma involving the spinal cord. An SCI can lead to a loss of function of the extremities, and the subsequent reduction in weight-bearing loads on these bones has been shown to cause osteoporosis through a variety of mechanisms. Two key components of mechanosensation include osteocytes embedded in the lacuna–canalicular system (LCS) and the presence of piezo receptors on the osteocytes themselves [60]. A protein called sclerostin is secreted by these osteocytes in the LCS at increased levels after a decrease in loading, which in turn antagonizes the canonical WNT/β–catenin signal cascade responsible for bone formation [60,61,62]. Additionally, inactivation of Piezo1 has been shown to decrease Wnt1 expression, a protein of the aforementioned canonical signaling pathway, which has been shown to lead to osteoporosis and fracture in animal models [60,63,64,65]. On the cellular level, apoptosis of both osteocytes and osteoblasts due to decreased loading have been demonstrated in murine models [66]. It seems that this apoptosis can be a triggering event for increased RANKL production [67], which can lead to an increase in osteoclastogenesis, although other studies have suggested that RANKL production and an increase in RANKL/osteoprotegerin (OPG) ratio can be seen independently of apoptosis in SCI models [21,68].

Polytrauma also may involve burn injuries, which can create long-lasting systemic inflammatory, stress, and hypermetabolic responses that may contribute to osteoporosis [2,69,70,71,72,73]. The inflammatory response is mediated by a cytokine reaction due to Toll-like receptors recognizing the molecular patterns of both endogenous cell damage and exogenous microbes entering due to the damaged skin barrier [73,74]. The stress response is proposed to be due to an increased release of glucocorticoids and catecholamines, and the hypermetabolic state due to the increased energy demand from the aforementioned inflammatory and stress responses [73]. The increased turnover of adenosine triphosphate (ATP) results in an increased energy demand, which can be supplied by muscle breakdown to the glucogenic amino acid alanine [75]. Additionally, the phosphate demand can be supplied by muscle breakdown, as it contains approximately 10% of the body’s phosphate [76]. The skeleton has a larger reserve of phosphate, and so this is a contributing factor to bone resorption in burn patients, although the primary mechanism is due to IL-1β and IL-6 from the systemic inflammatory response [73,76]. Delayed fracture healing was shown in a polytrauma murine model involving skeletal and burn trauma together, with a significant decrease observed in bone volume fraction throughout the defect area versus the osteotomy control [77]. In another mouse model of burn and skeletal trauma, significant cortical bone volume loss was reported compared to the cohort of skeletal trauma alone [3]. In a study of electrical burn patients, significantly decreased areal bone mineral density on dual X-ray absorptiometry (DEXA) scans was noted at L2–L4, the femoral neck, and total femur compared to the control [71]. These findings have been supported by a study of over 200,000 individuals, where a greater incidence of osteoporosis was found in the burn cohort compared to the control (IRR, 95% confidence interval: 1.35, 1.32–1.39) [2].

## 4. Risk Factors

Preventing additional fractures after a traumatic injury requires thorough risk assessment, followed by medical and lifestyle interventions. Nearly 50% of patients who survive a low-trauma osteoporotic fracture experience another fracture within 10 years [78], which is why it is critical to be aware of factors that increase the risk of post-traumatic osteoporosis. Several modifiable and non-modifiable risk factors exist and have been studied in both laboratory and clinical settings. These risk factors are similar to those seen in individuals at risk for osteoporosis in the general population but may be accelerated by a prior history of trauma.

Changes in remodeling: Disruption of bone microarchitecture is a primary risk factor for developing post-traumatic osteoporosis. Traumatic injury is known to disrupt the normal bone remodeling process, leading to increased bone resorption and decreased bone formation, which results in an imbalance and net loss of bone mass and density [79]. The structural integrity of bone is supported by a porous network and plates known as trabecular bone at the epiphyseal and metaphyseal plates [80]. The loss of this architecture results in a weaker structure, reduced mass, and increased risk of fracture [79]. This architectural weakness is a result of a disrupted balance between osteoclast-mediated dissolution and osteoblast-mediated bone formation [81]. Age-related declines in bone maintenance may further fuel this process [79]. Following traumatic injury, the absence of a mechanical load on bones, increase in metabolic stress, and endogenous and exogenous glucocorticoids are some of the factors that may initiate a disruption in the balance of bone remodeling [82,83]. Of note, patients with a prior history of fragility fractures (fracture from low-level trauma such as a fall from standing height) [84] have pre-existing osteoporosis with changes in bone remodeling that should not be attributed as a result of higher-level traumatic injury. However, these patients are described to have a significantly increased risk of imminent fracture within 1 and 2 years following the initial fracture as well as increased mortality and reduced mobility, which may be exacerbated by a more recent and higher-level traumatic injury [81,85]. This risk declines over time but remains higher than in patients who have not had fragility fractures [84].

Calcium/Vitamin-D: Deficiencies in calcium and vitamin-D have been well documented to promote bone resorption through increased osteoclast activity to maintain adequate serum calcium levels [86,87]. As such, low calcium and vitamin D may be significant risk factors for developing post-traumatic osteoporosis. In vitro rodent models have displayed disturbed fracture healing as a result of increased PTH and osteoclast activity, with an overall reduction in bone mass post-fracture [88]. Similarly, some studies have suggested that calcium is mobilized from bone deposits outside of the fracture site to compensate for low dietary calcium in order to successfully repair fractures [81,89]. Low vitamin D levels have been shown to alter normal calcium and phosphorus levels, cause secondary hyperparathyroidism, and reduce bone mineral density [90]. Each of these factors independently presents a risk for developing osteoporosis. However, evidence has also suggested contrary findings in patients without a prior history of fractures, known vitamin D deficiency, or osteoporosis. Supplementation with calcium and vitamin D3 in these patients, either alone or in combination, did not decrease fracture risk [91,92].

Age: The ability of bones to remodel and repair themselves becomes compromised with age, further exacerbating the risk for developing PTO [93]. Aging is associated with a natural decline in bone density and strength (osteopenia), which may progress to osteoporosis and place older individuals at increased risk for fractures [94]. In older adults, evaluations following a traumatic injury may reveal low bone mineral density (BMD) due to pre-existing osteoporosis [93,95]. As individuals age, there is a progressive decrease in bone mineral density, primarily affecting trabecular bone due to its higher metabolic activity and turnover rate compared to cortical bone [96]. This age-related decline in BMD is influenced by factors such as hormonal changes, decreased physical activity, and impaired remodeling capacity due to decreased osteoblast–osteoclast function [97]. Therefore, one possibility of older individuals presenting for evaluation after trauma is unmasking previously subclinical osteoporosis. Conversely older patients may develop new-onset osteoporosis as a result of increased metabolic stress, prolonged disuse or immobilization, and increased calcium mobilization due to the trauma. As previously described, calcium deposits in bone have been shown to mobilize in response to increased demand at a distant fracture site [81]. These changes are associated with low BMD and an increased risk of future fractures [98,99,100]. Low BMD as a result of trauma-related changes can lead to changes in trabecular architectural strength, such as decreases in trabecular volume and increases in the diameter of cavities and spacing within the matrix. This results in a loss of bone density and structural integrity, which increase susceptibility to future fractures, especially following the increased metabolic stress placed on the body following trauma [98]. Moreover, osteoblast activity has also been shown to decline with normal aging. This decreases efficacy of bone healing and regeneration after trauma and increases the risk of deregulated osteoblast–osteoclast remodeling, which together further increase the risk of PTO [101].

Postmenopause: postmenopausal women are particularly prone to developing osteoporosis after a traumatic injury. Osteoporotic fractures in this population are more common than stroke, myocardial infarction, and breast cancer combined [102]. A marked decrease in estrogen production surrounding menopause reduces the ability of estrogen to have its usual protective role in maintaining BMD [103]. The decline in estrogen levels coupled with aging contribute to accelerated bone loss and increased bone turnover, consequently leading to decreased BMD and increased fracture risk [103,104]. Pre-existing osteoporosis may be revealed during post-injurious evaluations of these patients. With postmenopausal estrogen decline, osteoclast activity is increased with a concurrent decrease in osteoblast activity [105] leading to an increased risk of fractures after trauma. In postmenopausal individuals without pre-existing osteoporosis, there may be an increased risk of developing PTO after a triggering injury. Both age-related and hormonal factors increase this risk, and the trauma-induced inflammatory response has itself been shown to increase bone resorption [106]. The imbalance between bone formation and resorption in a high metabolic state after trauma causes a net loss of trabecular bone, increasing susceptibility to fractures with a reduced healing capacity. Increases in follicle stimulating hormone (FSH) in postmenopausal women are a result of a loss of the negative feedback loop that estrogen exerts on pituitary FSH release. The combination of decreased estrogen and increased FSH has been shown to rapidly stimulate bone resorption, increasing the risk for more significant injuries and impaired healing after trauma [105]. Therefore, the additive effect of recovering from a traumatic injury superimposed on a postmenopausal state may increase the risk of developing PTO as well as increase the speed of development through a deregulation of homeostatic remodeling and healing [105,106,107]. A study by Leslie et al. demonstrated an association between patients with high-level trauma and an increased risk of future fractures among women with a mean age of 65 years [107]. Although postmenopausal women may be at increased risk for new-onset osteoporosis after trauma, the current literature focuses on the importance of screening for pre-existing osteoporosis when a postmenopausal woman presents with any type of fracture following high-level or low-level trauma [108].

Smoking: Cigarette smoke contains harmful substances that can negatively affect bone health, such as nicotine and toxins that disrupt the balance between bone formation and resorption, leading to accelerated bone loss [109]. Smoking has been shown to impair bone remodeling through the activation of NFκB, which controls inflammation and remodeling [109]. The activation of NFκB in osteoclasts leads to changes in gene expression that contribute to increased bone resorption [109]. There are also detrimental effects on wound healing and bone repair due to impaired bone vascular endothelium. This can increase post-traumatic complications such as avascular bone necrosis and disrupted recovery after injury [110,111]. Clinically, smokers are significantly associated with an increased risk of fractures compared to non-smokers [93]. The risk of any fracture is higher in current smokers, including adjustments for BMD [93]. The risk for vertebral body fractures and hip fractures is also elevated in current smokers, with a significantly higher risk for hip fractures [93].

Physical Activity: Patients who lack physical activity may be at an increased risk for PTO. Regular weight-bearing exercises (such as walking, jogging, or weightlifting, among others) stimulate bone remodeling and maintain BMD by placing mechanical stress on the bones [112,113]. Physical activity improves muscle strength and balance, which are essential to the prevention of falls and subsequent traumatic fractures. A lack of weight-bearing activities results in decreased BMD and compromised strength due to disuse and atrophy of bones and tissues [113]. Inactivity or prolonged immobilization, such as after a traumatic injury, can lead to bone loss through resorption, and this increases the risk for developing osteoporosis and future fragility fractures [113].

## 5. Role of Healthcare Providers in Reducing Risk

**Figure 2:**
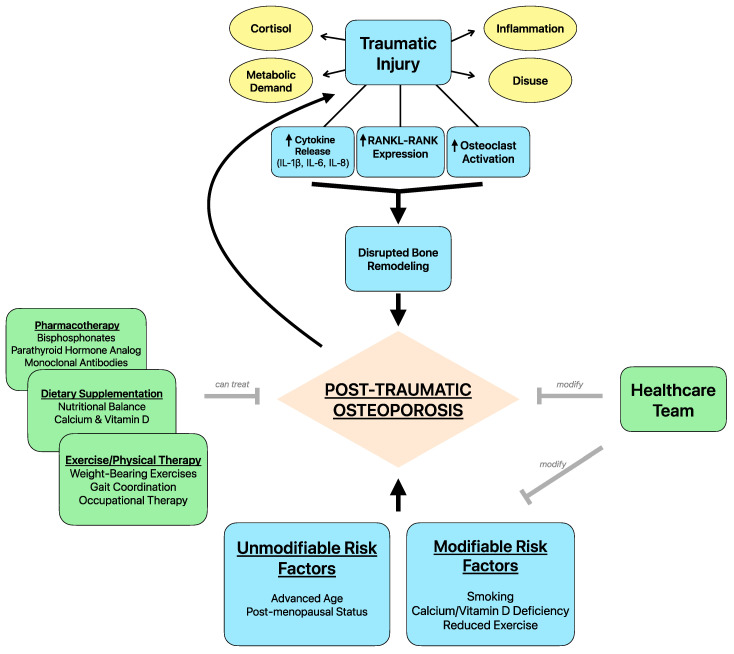
Schematic representation of post-traumatic osteoporosis.

It is crucial for every member of the healthcare team to possess comprehensive knowledge regarding the prevention, recognition, treatment, and management of both osteoporosis and related traumatic injuries, while also being mindful of the diverse backgrounds and needs of patients to provide optimal outcomes (Figure 2) [114]. An integrated healthcare approach is paramount to risk reduction, and assessing risk factors such as the degree of trauma sustained, family history of osteoporosis, and social determinants of health that may impact outcome and recovery. Management of comorbid conditions and mitigating hospital-acquired infections should remain a priority concurrent with the presenting acute injury. The current literature is limited regarding the role of healthcare providers in reducing the risk of PTO development in patients recovering from trauma. However, many strategies exist for improving patient adherence to therapy, patient education of sequalae following senile osteoporosis, and using an integrated healthcare approach to improve outcomes and ensure regular follow-up in these patients. Although not specifically studied, these strategies can be reasonably integrated into patients identified to be at risk of PTO.

Patient education is an important factor in the long-term care of patients with osteoporosis or those at risk of its development. A randomized trial by Gardner et al. described improved outcomes in patients whose osteoporosis care was followed beyond hospitalization. Patients were given an educational discussion during hospitalization, a list of questions to ask their primary physician, and a phone call reminder to follow-up within six weeks [115]. The study concluded that a multifaceted interdisciplinary approach towards patient education can significantly improve osteoporosis care by providing appropriate interventions, improved long-term care, and a decreased risk of recurrent fractures. Although this study did not specifically focus on patients recovering from traumatic injury, it may be beneficial for patients at risk for PTO to be provided with a similar education on their injury and the potential for developing osteoporosis. This approach may also improve awareness of PTO among patients and their primary care physicians, and could prompt discussion on concerning symptoms and osteoporosis screenings.

A multidisciplinary approach must be considered to decrease the risk of PTO. Improving medication adherence is critical to preventing fractures and improving outcomes. A review of interventions to improve medication adherence in osteoporosis patients found that motivational interviewing, counseling sessions on osteoporosis and its treatment, prompts or reminders for taking medication, and hospital-based osteoporosis coordinators are all promising methods to improving patient outcomes and adherence to therapy [116]. Though this review did not study a population of patients after trauma, the importance of adherence and an integrated patient-centered team approach are highlighted for patients with senile osteoporosis. Because of this, it may be reasonable to achieve similar rates of improved adherence and education in trauma patients.

Depending on the institution and local regulations, pharmacists may play a unique role in improving osteoporosis outcomes. A multinational review by Elias et al. found that pharmacist interventions such as counseling and education on medication use, adherence to therapy, and the importance of calcium, vitamin D, and exercise in high-risk individuals improved osteoporosis screening and calcium intake among these patients [117]. While this review did not specifically focus on patients following trauma, pharmacists may be able to play a unique role in improving PTO screening and adherence to therapy in trauma patients as well. Further studies are warranted to explore this.

The presence of a dedicated osteoporosis coordinator has been shown to improve post-fracture osteoporosis management through individualized case management. Fracture Liaison Services (FLSs), also known as osteoporosis coordinator programs, provide a systematic approach for the assessment and management of postmenopausal women and older men who have sustained low-trauma fractures [118]. These programs enable healthcare providers to identify individuals at risk of osteoporosis and implement appropriate interventions to prevent further fractures. Coordinators are responsible for assessing patient fracture risk, providing education, referring patients for follow-up visits, and recommending BMD imaging assessments [78]. These programs aim to fill the care gap in at-risk patients with fractures who were not diagnosed with osteoporosis or those who did not receive or seek treatment [78]. Studies on osteoporosis coordinators and FLSs were not specifically focused on populations of trauma patients. However, given the clear benefits in senile traumatic osteoporosis patients, the use of coordinators and FLSs specific to trauma patients may provide benefits in screenings and assessments in at-risk patients.

Healthcare teams can play a critical role in the identification and screening of trauma patients at risk for PTO. Although current studies do not specifically explore the role of healthcare providers in reducing osteoporotic risk in trauma patients, providers may use their judgement to implement strategies that have been shown to be efficacious in patients with senile osteoporosis. Further studies should be conducted to determine the best practices for a partnered approach in PTO risk reduction.

## 6. Symptoms and Diagnosis

Osteoporosis frequently remains asymptomatic until a patient experiences a pathologic fracture from low force, minor trauma, or daily activities [84]. In the rare cases when the osteoporosis is symptomatic, severe pain at rest is characteristic of the disease. When osteoporotic fractures do occur, they often impact the hip, vertebral bodies, and distal forearm [84]. Frequent osteoporotic fractures increase morbidity and mortality while also diminishing quality of life, and they should warrant further investigation in patients who have recently sustained traumatic injury.

Osteoporosis is a condition that shares many qualities with and oftentimes mimics other musculoskeletal pathologies, and thus providers ought to rule out other pathologies prior to beginning osteoporosis treatments. One of these conditions is osteomalacia—a disorder in which vitamin D deficiency and impaired mineralization can produce skeletal deformities and pain [119]. However, an important distinction is that osteomalacia is characterized by a low bone mineral to matrix ratio while the ratio is preserved in osteoporosis. Further clinical evaluation into pathologic fractures must rule out metastatic bone disease before osteoporosis can be diagnosed. Osteolytic metastases, characterized by the destruction of the bone, are a known cause of pathologic fractures, namely in long bones [120,121]. Additionally, hyperparathyroidism can cause mineral release from the bone, predisposing patients to pathologic fractures that may initially mimic osteoporosis until proven otherwise [122]. Understanding the myriad of conditions that may appear similar to osteoporosis, thorough work-up and exclusion of these criteria—along with consideration of recent skeletal trauma—remains an important step in its eventual diagnosis.

**Figure 3:**
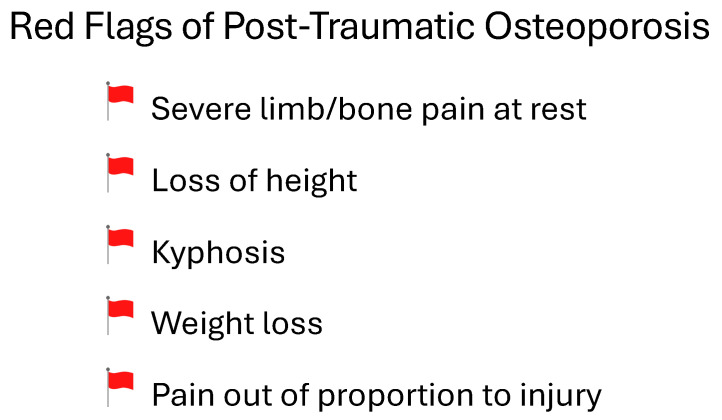
Red flags of post-traumatic osteoporosis.

Several symptoms—so called “red flags”—may clue providers into a case of PTO and warrant further work-up (Figure 3). Severe bone pain at rest is seen in osteoporosis, which is thought to be due in part to an increased sensory fiber density [123]. In patients with PTO who have progressed to osteoporotic spinal fractures, a loss of height and kyphosis may be seen [124]. Weight loss due to decreased BMD, caused by decreased mechanical loading and increased calcium mobilization [125], may also be a clinical sign of PTO, although a non-specific one [126]. Finally, pain out of proportion to injury, typically dull back pain as a sequela of pathologic vertebral fracture, can be seen [127].

Osteoporosis is primarily characterized by bone strength deterioration due to microarchitecture deterioration—resulting in mechanical failure and pathologic fracture. Formal evaluation for osteoporosis involves bone densitometry tests and dual-energy X-rays [128]. Lower results on densitometry tests (less than 2.5 standard deviations below the population mean) indicate osteoporotic areas. Further evaluations of osteoporosis can involve measurements of urinary N-telopeptide [129] and normal laboratory values of phosphorus, calcium, vitamin D, parathyroid hormone, and alkaline phosphatase. In certain cases when dual-energy X-rays are inappropriate, quantitative ultrasound may be employed to evaluate for osteoporotic bone change—namely in the calcaneus and radius [130]. Although the pathogenesis of osteoporosis is multifactorial, bone density measurements are considered to be a strong predictor of future fracture and eventual complications [128]. When evaluating for osteoporosis after injury or trauma, considerations of serial bone densitometry exams in the affected region might indicate a new onset of osteoporosis.

## 7. Treatment and Management

The management of osteoporosis following traumatic injury follows similar guidelines to osteoporosis treatment in the general population. First-line treatment should focus on rehabilitating the causative injury followed by a focus on osteodegeneration. A baseline bone mineral density assessment obtained through dual-energy X-ray absorptiometry (DEXA) may aid follow-up care and help track recovery [115].

Beyond care of the acute injury, three broad categories of management are regularly seen in the current literature: pharmacotherapy, dietary management, and physical rehabilitation of musculoskeletal health. The following studies and management strategies are not specific to patients following trauma, but they do include the management of osteoporosis in trauma patients.

Pharmacotherapy involves basic treatment with 1000 IU per day of vitamin D3 and 1000 mg calcium per day through supplements, although a dietary intake is preferred. In general, anti-resorptive agents reduce bone turnover and formation stimulators support bone deposition. Oral bisphosphonates such as alendronate and risedronate are typically first-line therapies for osteoporosis. They inhibit bone resorption by osteoclasts and may be taken if patients are able to sit up straight for 30 min, have intact renal function (GFR > 30 mL/min), and do not have gastroesophageal disease [131]. Hospitalized patients on proton pump inhibitors such as omeprazole may not benefit from oral bisphosphonates due to decreased absorption; additionally, combining bisphosphonates with PPIs may increase fracture risk [132]. Intravenous bisphosphonates such as ibandronate and zoledronate may be used in this case, the latter of which has been shown to increase BMD and significantly reduce fractures and mortality in a population of patients with traumatic hip fractures [131,133]. Parathyroid hormone analogs such as teriparatide induce mineral deposition and effectively increase BMD by stimulating osteoblasts. Patients without contraindications (hypercalcemia, renal failure, Paget’s disease, or hyperparathyroidism) may be administered daily subcutaneous injections for a maximum of 2 years. Monoclonal antibodies such as denosumab can be used to inhibit bone resorption and are safe for use in patients with renal deficiency. Denosumab is a monoclonal antibody to RANK-L and functions by inhibiting the binding of RANK-L on osteoclasts to the RANK receptor on osteoblasts, thereby reducing bone loss [134]. Romosozumab is a monoclonal antibody sclerostin inhibitor that stimulates osteoblasts to increase bone formation and decrease bone resorption [135]. Lastly, although selective estrogen receptor modulators (SERMs) and hormone replacement therapy have been successful in treating osteoporosis in postmenopausal women, they may be less useful after trauma due to the risk of thrombosis [131].

The dietary management of trauma patients at risk for osteoporosis must focus on nutritional support to meet the increased metabolic demand following trauma. Malnutrition has been identified as an independent risk factor for complications, mortality, prolonged hospital length of stay, and declined quality of life in severely injured trauma patients [136]. Early enteral and parenteral nutrition have been shown to improve outcomes by providing essential macro- and micronutrients. The treatment of underlying serum deficiencies with increased supplementations of calcium and vitamin D has also been shown to strongly influence bone mass [137]. Smoking cessation, increased protein intake, and decreased intakes of alcohol, caffeine, and sodium have also been shown to be protective from osteoporosis [138]. Improving nutritional balance in this way may decrease the risk for PTO and improve healing time.

Improvement of physical function and rehabilitation from the causative traumatic injury among PTO patients is vital in maintaining a lower long-term osteoporotic fracture risk. Physical therapy for strength improvement and weight-bearing exercise can improve the balance and coordination of trauma patients, placing them at a lower risk for future fractures. Flexibility and posture training are particularly important due to the high incidence of vertebral fractures in patients with osteoporosis [139]. Occupational therapy to assist in performing activities of daily living has been demonstrated to reduce the occurrence of future fractures after hospitalization. In trauma patients, the focus should be placed on strengthening the injured bone or extremity, as early weight bearing has been shown to improve strength and outcomes in lower-limb fractures [140]. The safe mobilization of fractured bones following trauma plays a crucial role in improving mobility, strength, and preventing disuse-related complications. Early mobilization and rehabilitation strategies are essential in recovery from trauma, as these strategies may reduce the risk of long-term complications such as osteoporosis. Healthcare providers should caution against excessive weight or force to protect patients against unintentional injuries from premature healing. By ensuring safe mobilization and encouraging the use of fractured bones through appropriate rehabilitation protocols, healthcare providers may promote better outcomes for patients post-trauma.

## 8. Prognosis and Follow-Up

The primary goal in treatment and the strongest prognostic factor in osteoporosis is avoidance of fractures. If fractures have already occurred, the secondary treatment objective is the prevention of further fractures. In patients who have already experienced fractures, prognosis is largely influenced by the specific anatomical location of fracture. As most fractures tend to occur in the hip and vertebrae [141], patients might experience limits in mobility and physical activity. While osteoporosis itself might not severely impact a patient’s prognosis, the limitations and disabilities that come from the fractures might do so. Resultantly, studies have shown that life expectancy in individuals with osteoporosis can be significantly less than those without the condition. Abrahamsen et al. have described an excess mortality risk in those treated for osteoporosis when compared to the background population—with the risk being notably higher within the first few years of treatment [142]. While further literature on the prognosis remains scarce, individuals who are diagnosed early and started on adequate treatment can live for three to four decades following a diagnosis and can maintain an adequate quality of life.

Individuals living with osteoporosis can improve their prognosis and quality of life through personalized rehabilitation plans. Regardless of the anatomical region fractured, a comprehensive rehabilitation plan that includes education, strength training, and the prevention of further fractures can be beneficial to patients of both idiopathic and post-traumatic osteoporosis [143]. Specific strengthening exercises have been shown to be effective in preventing re-fracture and improving the quality of life in both osteoporotic hip and vertebral fractures [144].

Despite the presence of guidelines for follow-up care from organizations such as the National Osteoporosis Foundation (NOF), the American College of Physicians (ACPs), and the International Osteoporosis Foundation (IOF), challenges may exist in their implementation or adherence in clinical practice [145]. Understanding this, however, patients with osteoporosis require more frequent doctor appointments, lab visits, and medication monitoring to ensure their symptoms are managed. Further, living with osteoporosis might warrant repeat bone density scans and routine endocrinology visits [146]. At follow-up visits with providers, more attention might be focused on primary prevention of osteoporotic fractures and managing lifestyle choices/expectations. With these visits and the appropriate treatment, individuals with all forms of osteoporosis can optimize their health outcomes.

## 9. Future Directions

This comprehensive literature review has exposed several critical knowledge gaps concerning osteoporosis secondary to trauma. As it stands, there is considerable research on the underlying dysregulation of cytokines, hormones, and vitamins secondary to various traumatic events. Additionally, there is substantial evidence of the effect of these mediators on the bone microenvironment (Figure 1). However, key translational research bridging these two bodies of evidence is lacking.

Furthermore, as PTO may be masked by the general declining health of patients affected by trauma and can be misdiagnosed or misattributed by providers, there is a lack of clinical research. Despite the challenges of identifying and assessing patients with this condition, which are compounded by the presence of complex patients with multiple comorbidities, it is imperative to conduct clinical research to safeguard the skeletal integrity and overall well-being of these individuals.

There is a notable absence of outcome data regarding treatment modalities for post-traumatic osteoporosis. While there is literature describing various physical and pharmacotherapeutic interventions for post-traumatic osteoporosis, it is important to note that the evidence regarding their efficacy and long-term outcomes may vary. Some studies have reported outcome data for treatments such as bisphosphonates, parathyroid hormone, or estrogen modulator therapy following traumatic injuries. However, there may still be gaps or inconsistencies in the available evidence, and further research is warranted to establish their effectiveness conclusively. Moreover, the prevailing literature predominantly focuses on older adults, leaving a conspicuous void in the knowledge regarding the enduring consequences of post-traumatic osteoporosis in individuals under 18 years old. Furthermore, the impact of non-pharmacological interventions such as exercise and nutrition remain inadequately explored.

## 10. Limitations

In addressing the limitations of our study, it is essential to acknowledge several key points. Firstly, while we have aimed to provide a comprehensive overview of PTO, it is important to recognize that our review may not have captured every relevant study or perspective on the topic. Secondly, the quality and heterogeneity of the included studies may have impacted the robustness of our conclusions. The variability in study designs, patient populations, and outcome measures across the literature may have introduced bias or limited the generalizability of our findings. Additionally, the reliance on existing literature means that our review is subject to the limitations inherent in primary studies, including potential methodological flaws, incomplete reporting, and variability in data quality. Finally, our review primarily focused on summarizing existing evidence rather than generating new data. As such, while we aimed to identify and critically evaluate the available literature, our review may not have addressed all of the potential research questions or explored alternative perspectives on the topic.
